# Supplementary material for: Opening the Black Box of Imputation Software to Study the Impact of Reference Panel Composition on Performance
Source: Genes (Basel). 2023 Feb 4;14(2):410. doi: 10.3390/genes14020410 (PMC9956390; doi:10.3390/genes14020410)
Supplement: Supplementary file 1 [file genes-14-00410-s001.zip › genes-2149329-supplementary.pdf]

Opening the black-box of imputation software to study the impact of reference panel composition on performance

Supplementary Figures and Tables

T Dekeyser[1,2], E Génin[1,2], AF Herzig[1,*]

1 : Inserm, Univ Brest, EFS, UMR 1078, GGB, Brest, France
Faculté de Médecine - IBRBS 22 avenue Camille Desmoulins F-29238 BREST Cedex 3 - France
2 : CHRU Brest, Brest, France
* Corresponding Author

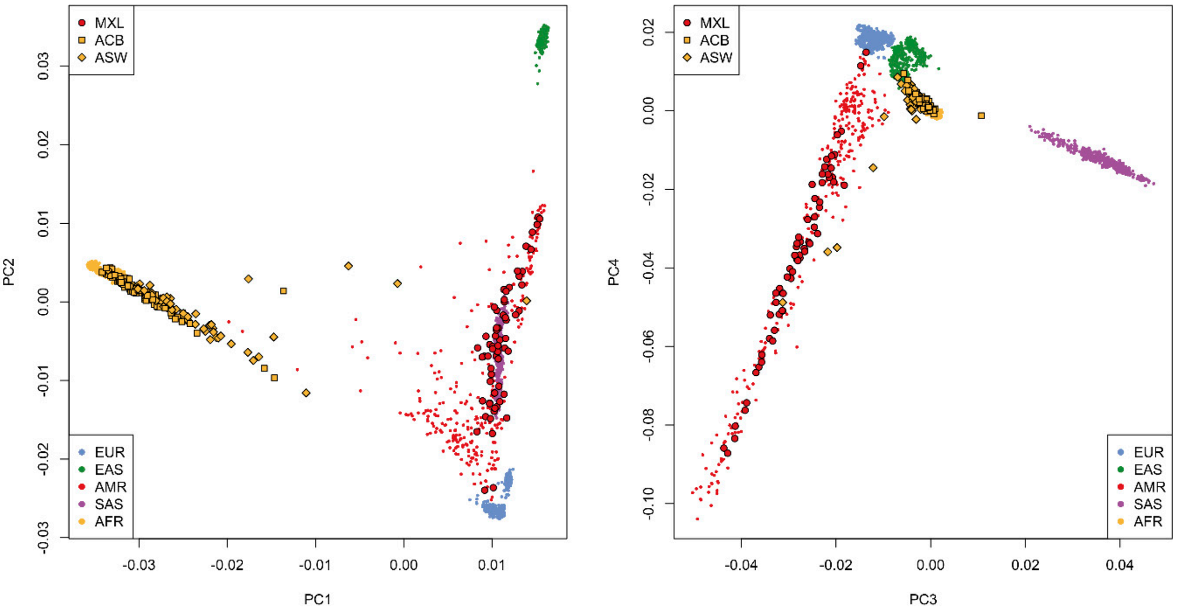

Figure S1. Principal components analysis of the 1000 Genomes project individuals. The individuals of our target group (ACB, ASW, and MXL) are highlighted. Colours refer to super-population attribution (see Table S1).

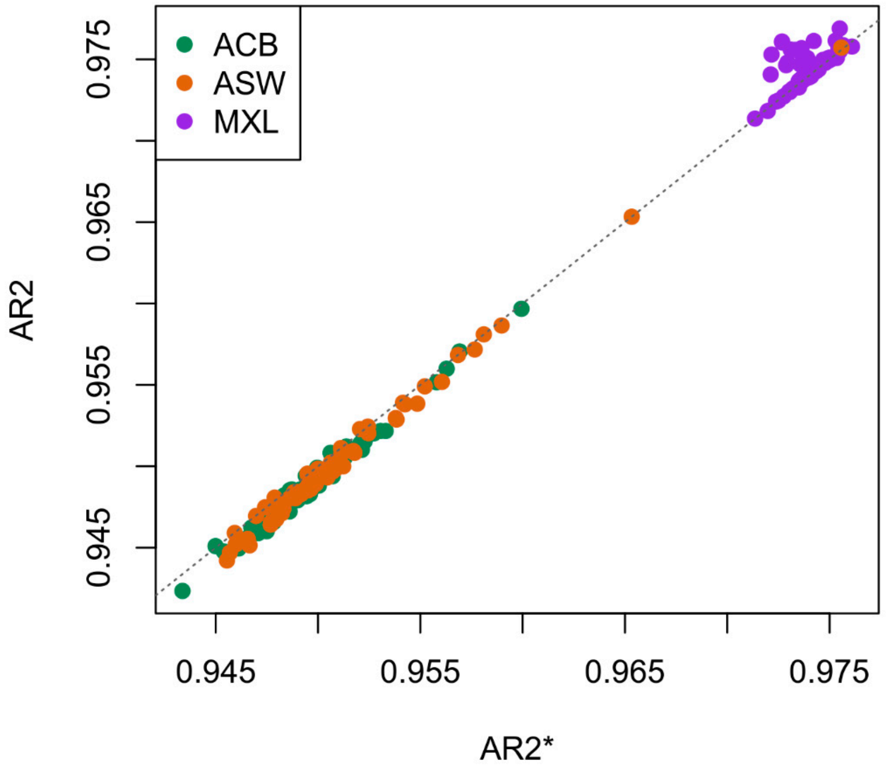

Figure S2. Individual aggregate R^2 (AR2) (IMPUTE5 with all populations) and AR2* (IMPUTE5 with SOURCEFIND populations) statistics that correspond to those presented in Figure 2 in the main text.

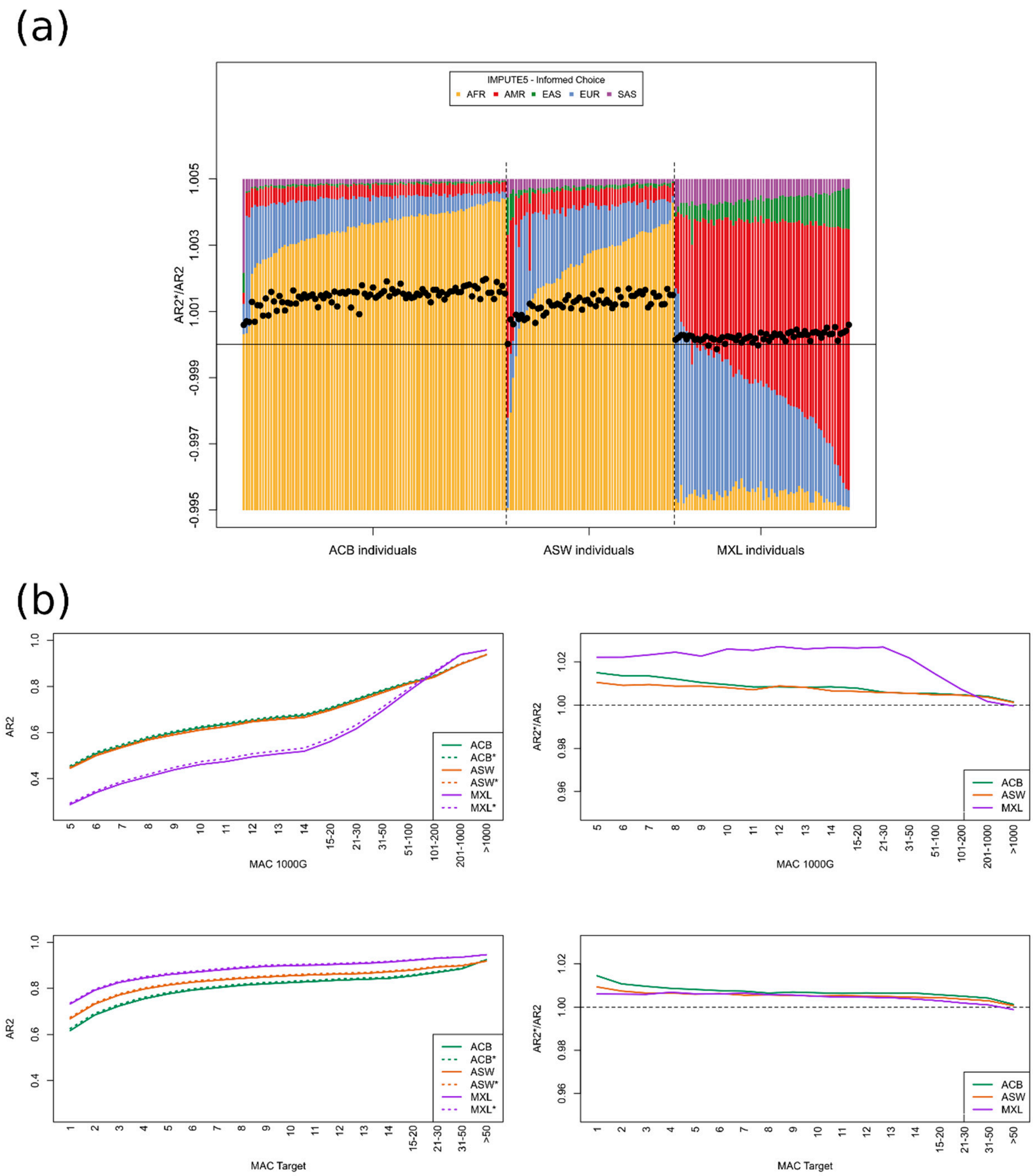

Figure S3. This figure echoes Figure 4 in the main text. The admixture plot in (a) shows the cumulative imputation barcodes used by IMPUTE5 under the informed choice approach; exactly as in Figure 4. The AR2*/AR2 statistics overlaid in (a) and given in more detail in (b) however correspond to imputation using MINIMAC4.

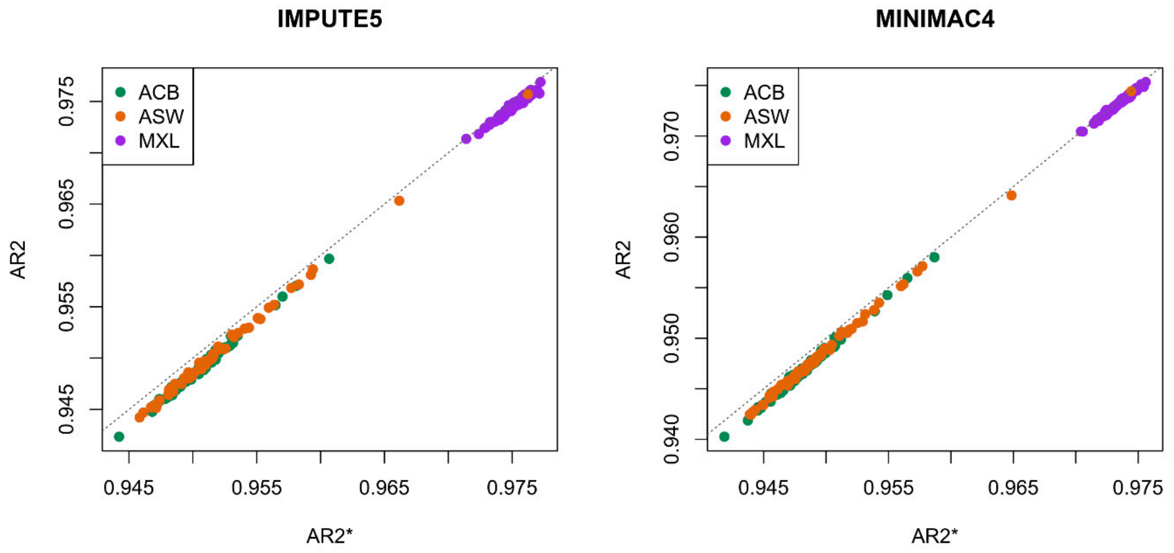

Figure S4. As Figure S2 but pertaining to the 'informed choice' strategy and showing the individual aggregate R^2 statistics from Figure 4 in the main text for IMPUTE5 and Figure S3 for MINIMAC4.

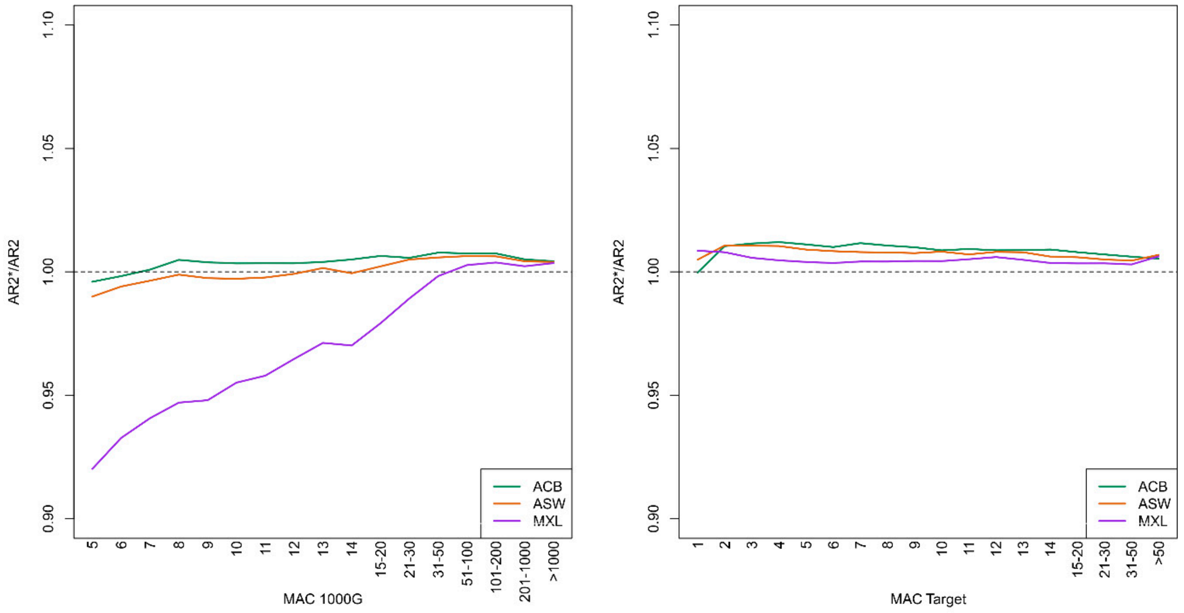

Figure S5. Comparison of IMPUTE5 and MINIMAC4. The ratio AR2*/AR2 describes the ratio of aggregate R^2 statistics from IMPUTE5 (AR2* for this plot) against MINIMAC4 (AR2 for this plot). Values above or below the horizontal line at 1 indicate that the imputation was more or less accurate with IMPUTE5; respectively. Results are split by minor allele count (MAC), either calculated in the entirety of the 1000G (left) or within each of the three target groups (right).

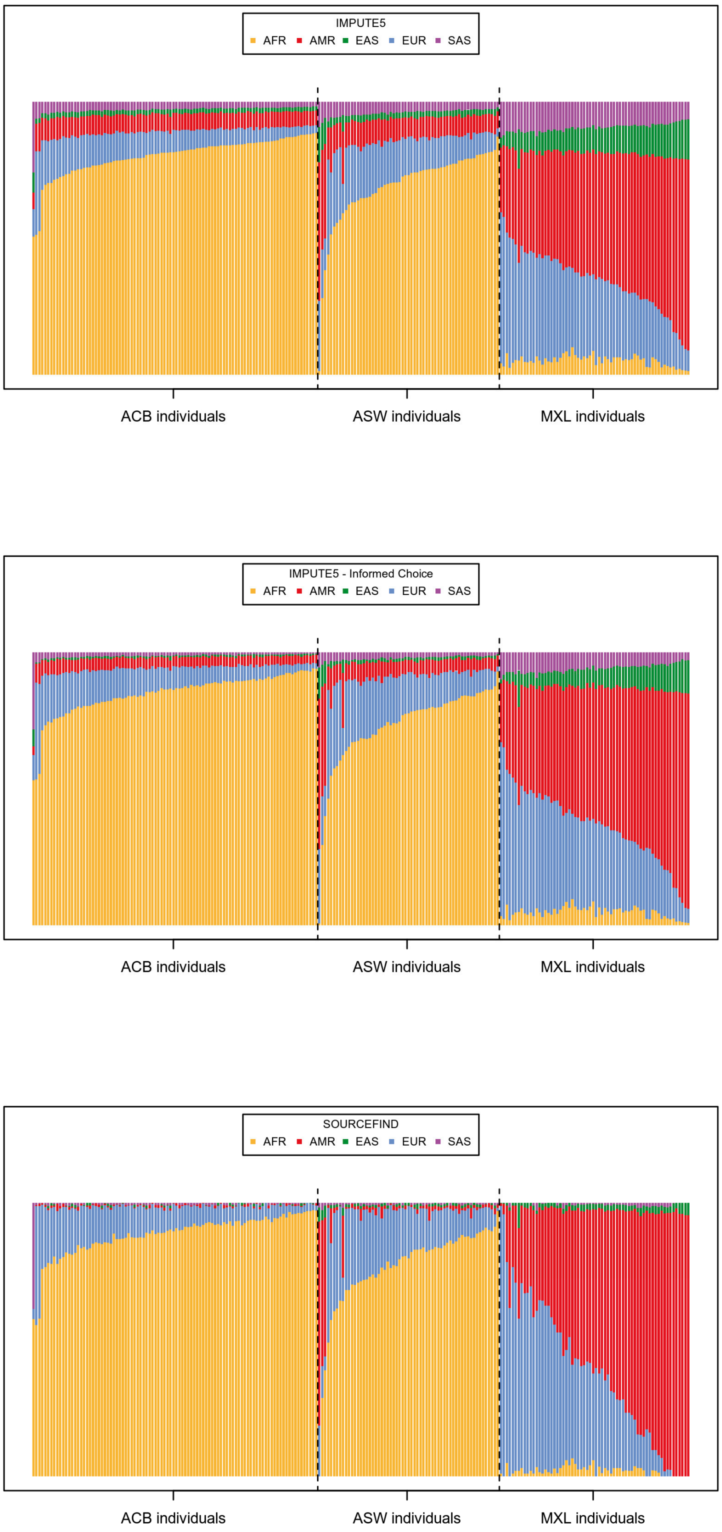

Figure S6. Proportions of the genome imputed with different populations when using IMPUTE5 with the complete 23 population reference panel (top), with the informed choice strategy (middle), or the proportions as were estimated by SOURCEFIND (bottom). The top and bottom panels appear in Figure 2 in the main text, and the middle panel appears in Figure 4 in the main text, they are put together here to facilitate their comparison.

Super Population	Populations
AFR (Africa)	LWK, GWD, MSL, **ACB, ASW**, YRI, ESN
EAS (East Asia)	CHB, KHV, CHS, JPT, CDX
EUR (Europe)	TSI, CEU, IBS, GBR, FIN
SAS (South Asia)	BEB, STU, ITU, PJL, GIH
AMR (Americas)	PEL, **MXL**, CLM, PUR

Table S1. The populations of the 1000 Genomes projects, split into five super-populations. Three populations (in bold) were chosen to be our target individuals: ACB (African Caribbean in Barbados), ASW (African Ancestry in South-West USA), and MXL (Mexican Ancestry in Los Angeles California USA). In this study, the 221 individuals from these three groups were imputed, in a variety of ways, using the other 23 populations as a reference panel.

Hard-called dosage →		MINIMAC4			MINIMAC4, informed choice		
Truth ↓		AA	Aa	aa	AA	Aa	aa
MAC 0	AA	1438349128	570847	417	1438383014<br>+ <0.01%	536997<br>-5.93%	381<br>-8.63%
	Aa	-	-	-	-	-	-
	aa	-	-	-	-	-	-
MAC 1	AA	626859413	511378	349	626879224<br>+ <0.01%	491580<br>-3.87%	336<br>-3.72%
	Aa	1060454	1789942	1291	1056408<br>-0.38%	1794103<br>+0.23%	1176<br>-8.91%
	aa	-	-	-	-	-	-
MAC 2	AA	466653082	477660	331	466671271<br>+ <0.01%	459495<br>-3.80%	307<br>-7.25%
	Aa	1400943	2841075	2196	1394398<br>-0.47%	2847745<br>+0.23%	2071<br>-5.69%
	aa	2260	3754	4848	2213<br>-2.08%	3883<br>3.44%	4766<br>-1.69%
MAC 3	AA	343451450	426792	326	343467622<br>+ <0.01%	410666<br>-3.78%	280<br>-14.1%
	Aa	1412081	3275216	2998	1407515<br>-0.32%	3279980<br>+0.15%	2800<br>-6.60%
	aa	3533	7084	10229	3444<br>-2.52%	7227<br>2.02%	10175<br>-0.53%

Table S2. This table echoes Table 1 in the main text but the results here correspond to MINIMAC4 and not IMPUTE5.
